# Ectopic bronchogenic cyst arising from the diaphragm: a rare case report and literature review

**DOI:** 10.1186/s12893-021-01317-w

**Published:** 2021-08-10

**Authors:** Juan Tang, Zhen Zeng, Senyi Deng, Feng Lin

**Affiliations:** Department of Thoracic Surgery, West China Hospital, Sichuan University, No. 37, Guoxue Alley, Sichuan 610041 Chengdu, China

**Keywords:** Case report, Bronchogenic cyst, Thoracoscopic surgery

## Abstract

**Background:**

Bronchogenic cysts can be caused by errors in the growth of the ventral foregut. Localization of the bronchogenic cyst (BC) varies depending on the level of the abnormal budding. They are usually located in the lungs and mediastinum. BCs of the diaphragm are a rare form of this abnormality.

**Case presentation:**

A 66-year-old woman coughs and expectorates. CT scan evaluation revealed a soft tissue shadow of 6 × 5 cm in the left lung. Under thoracoscopic surgery, we found that the mass originated from the diaphragm away from the lung tissue, we completely removed the mass and the pathological result was diagnosed as BC.

**Conclusions:**

The prognosis of ectopic BC is usually optimistic for benign tumors, as long as the tumor is completely removed.

## Background

Bronchogenic cysts (BCs) are congenital malformations of the ventral foregut, caused by abnormal sprouting of the original tracheobronchial tree. They are usually located in the mediastinum, lungs, and other rare places, depending on the level of abnormal budding that occurred during development [1]. Occurrence of bronchogenic cyst in the diaphragm is extremely rare. Here, we report a case of ectopic bronchogenic cyst arising from the diaphragm in an adult. This case was confused with a lung tumor before operation at radiological imaging. Eventually, the patient underwent successful minimally invasive surgery with a smooth recovery.

## Case presentation

**Fig. 1:**
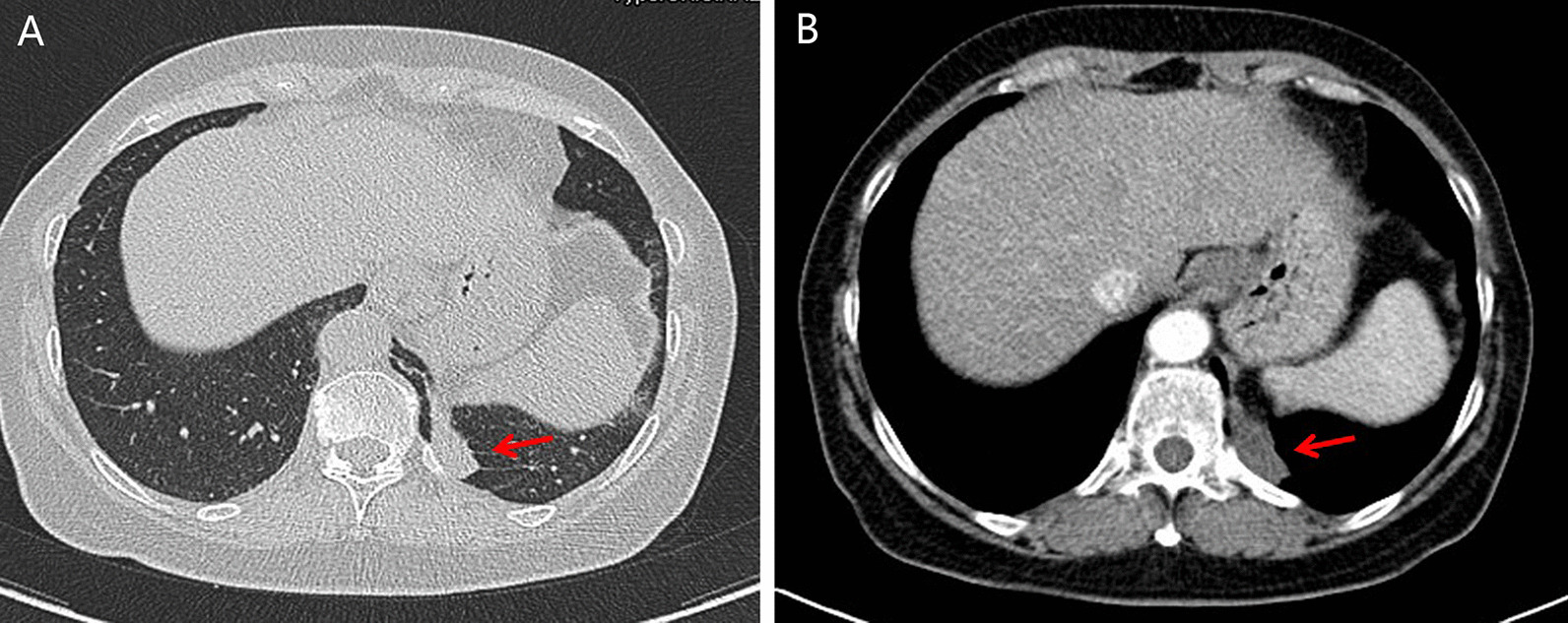
**A**, **B** Computed tomography (CT) of chest with contrast showed a 6 × 5 cm soft tissue in the posterior basal segment of the left lower lobe of the lung, and its adjacent pleura is thickened (arrows)

**Fig. 2:**
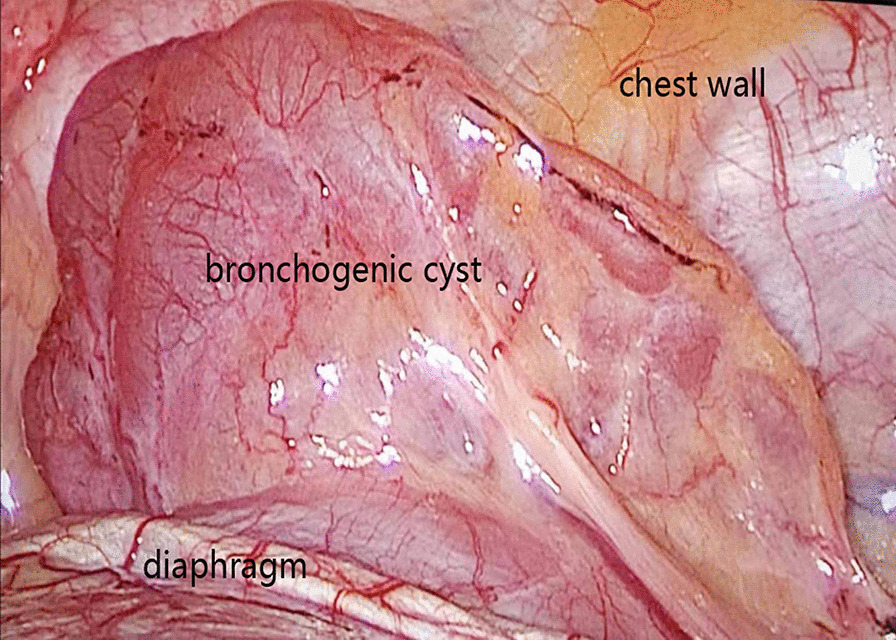
During operation, a 6 × 5 × 2 cm lamellar cystic tumor arising from the diaphragm, adjacent to the chest wall, was found (arrow)

**Fig. 3:**
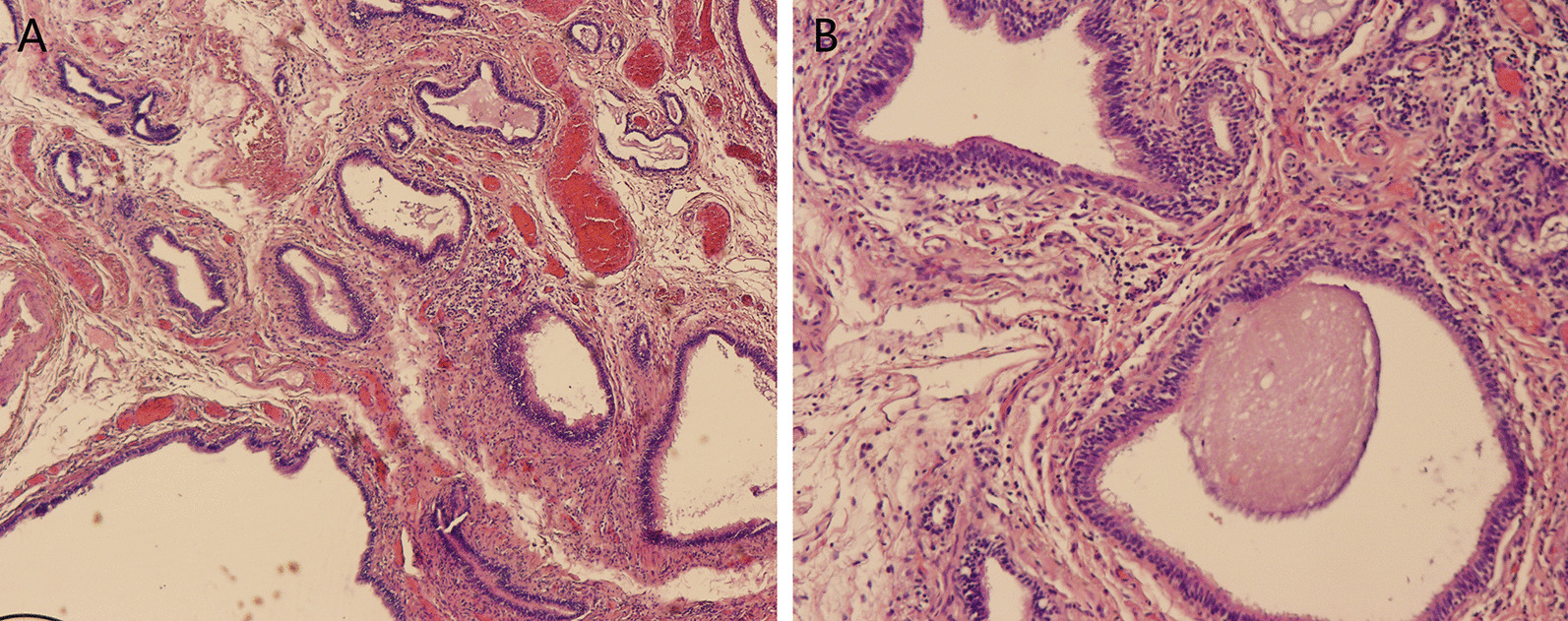
Histopathologic examination indicated the cyst was composed of smooth muscle, loose connective tissue, and pseudostratified ciliated columnar epithelium without cellular atypia (Haematoxylin and eosin stain, **A** original magnification × 4, **B** original magnification × 10)

A 66-year-old woman presented with a 6-month history of cough with blood in the sputum. The patient was treated with antibiotics without significant relief of symptoms by pulmonologist. She denied fever, chest pain and tightness. Clinical history and family history were uneventful. Physical examination revealed all vital signs were normal. The laboratory data revealed a slightly elevated folate receptor-positive circulating tumor cells count of 14.0 FU/3mL. Then, a computed tomography (CT) of chest with contrast was performed, which showed a 6 × 5 cm soft tissue in the posterior basal segment of the left lower lobe of the lung, and its adjacent pleura was thickened (Fig. 1 A, B, arrows). Since the diagnosis of the tumor could not be ruled out, the patient underwent an exploratory video-assisted thoracic surgery (VATS) with general anesthesia. During operation, a 6 × 5 × 2 cm lamellar cystic tumor arising from the diaphragm, adjacent to the chest wall, was found (Fig. 2) and the cystic tumor was completely resected through linear cutting stapler. Histopathologic examination indicated a cystic structure filled with thick white mucus (Fig. 3). Microscopically the cyst was composed of smooth muscle, loose connective tissue, and pseudostratified ciliated columnar epithelium without cellular atypia. After operation, he was given routine antibiotics to prevent infection for 2 days. The patient was discharged after an uneventful postoperative recovery.

## Discussion and conclusions

BC is a kind of rare benign tumor originated early in lung bud development before bronchus formation. Clinically, BCs are usually located in the lung parenchyma or mediastinum [2, 3], but in some case they may break away and migrate to other locations [4, 5]. Therefore, BCs can be divided into three types: intrapulmonary, mediastinal and ectopic, according to the location of lesions [6]. Of these, the incidence of ectopic type is the lowest. Usually, most patients have no or minimal clinical symptoms caused by compression of surrounding structures or complications related to cysts [5].

Ectopic BCs are extremely uncommon. The literature review (Table 1) reveals that few cases of ectopic BCs have been reported in the past 15 years [1–24]. The most common sites are neck, thyroid, stomach, esophagus, scapular, parietal pleura and pericardium. Rare cases occurring in diaphragm have also been reported [19, 20]. As for ectopic BC in diaphragm mimicking lung tumor similar to our report, only 1 cases have been reported in the English literature up to now [25] and 2 cases in another language [26, 27]. Usually, there is a direct correlation between clinical symptoms and lesion sites [13], and the patients of ectopic BCs may present a series of symptoms such as abdomen pain (stomach), dysphagia (esophagus),dyspnea (neck) and chest pain (pericardium). In addition, there are some potential complications of BC including infection, rupture of the cyst, bleeding, and even malignant transformation [2, 14].

**Table 1:** The reported case of ectopic bronchogenic cyst in the past 15 years

N	Author	Age	Sex	Location	Size (cm)	Symptom	Treatment	Follow-up
1	Mir ZM [1]	5	F	Scapular	3.9 × 2.9 × 3.7	Cellulitis	Surgical resection	No recurrence
2	Cheng Y [2]	30	M	Esophagus	4.0 × 7.0 × 8.0	Dysphagia	Surgical resection	No recurrence
3	Kün-Darbois [3]	0.25	F	Tongue	d = 1.0	Swelling of tongue	Surgical resection	No recurrence
4	Sang YS [4]	62	F	Stomach	d_=_1.6	None	Surgical resection	No recurrence
5	Chhaidar A [5]	65	F	Stomach	8.0 × 7.0	Epigastric pain	Surgical resection	No recurrence
6	Xiao J [6]	62	F	Stomach	7.0 × 4.5 × 1.5	Abdomen pain	Surgical resection	No recurrence
7	Usamah M [7]	7	M	Neck	4.0 × 3.0	None	Surgical resection	No stated
8	Ustundag E [8]	5	F	Thyroid	2.0 × 2.0	Swelling	Surgical resection	No stated
9	Somwaru LL [9]	3	F	Intrapericardial	4.0 × 2.0	No stated	Surgical resection	No stated
10	Nakagawa M [10]	1.25	F	Parietal pleura	2.1 × 1.8	Fever	Surgical resection	No stated
11	Petraud A [11]	22	M	Tongue	d = 2.0	Painful	Surgical resection	No stated
12	Al-Balushi Zi [12]	3	M	Scapular	4.0 × 3.5 × 2.0	None	Surgical resection	No stated
13	Lu Q [13]	46	M	Pericardium	10.0 × 8.0 × 7.0	Dyspnea, chest pain	Surgical resection	No recurrence
14	Sun L [14]	67	M	Stomach	d = 5.0	Epigastric pain	Surgical resection	No recurrence
15	Kiralj A [15]	6	F	Neck	3.5 × 4.0	Dyspnea	Surgical resection	No recurrence
16	Liu Z [16]	70	F	Neck	3.3 × 3.0	Painful mass	Surgical resection	No recurrence
17	Yang X [17]	40	M	Esophagus	3.0 × 2.0	None	Surgical resection	No recurrence
18	Xiang J [18]	23	M	Esophagus	4.5 × 3.8	Dysphagia	Surgical resection	No stated
19	Mubang R [19]	41	M	Diaphragm	4.5 × 5	Back pain	Surgical resection	No stated
20	Jiang C [20]	38	F	Diaphragm	5 × 5 × 4	None	Surgical resection	No recurrence
21	Shah SK [21]	1.9	F	Subcutaneous	d = 1.5	Fluctuant mass	Surgical resection	No stated
22 23 24	Borgnat F [22] Parambil JG [23] Kubouchi Y [24]	5 38 19	N/A M M	N/A Mediastinum Mediastinum	N/A 4.4 × 2.4 × 4.5 3.5 × 2.0	N/A Atrial fibrillation None	Surgical resection Surgical resection Surgical resection	No stated No recurrence No stated

Radiological examination has great clinical value for the early discovery of ectopic BCs [10]. However, due to the lack of specific manifestations at CT or magnetic resonance imaging (MRI) [28], it is easy to be confused with common primary organ disease, and the misdiagnosis rate of this disease may reach 40 to 60 % [6]. In some literature reports, ectopic BCs have been confused with peripheral lung neoplasm, diaphragmatic tumor, diaphragmatic hernia, neurogenic tumor, hydatid cysts, and esophageal diverticulum [20]. In our case, the patient was initially diagnosed with a lung tumor due to respiratory symptoms and atypical CT images. She had been treated with conservation therapy but did not relieve. A complete excision was considered to be an effective therapeutic strategy. The cyst arising from the diaphragm was not found until the operation, and the pathological examination confirmed the diagnosis of ectopic BC.

Considering the potential risk of complications and malignant transformation, complete surgical resection is recommended even for asymptomatic patients [5]. Minimally invasive surgery, such as VATS, is a valuable diagnostic method to identify the location of lesions and then help diagnosis [2]. In some complex cases, transtracheal and percutaneous cyst aspiration has been proposed as an alternative to surgery, but it has not been widely accepted due to possible high incidence of recurrence. In general, the prognosis of ectopic BC is usually optimistic for benign tumors, as long as the tumor is completely removed [29].

## Data Availability

Not applicable.

## Abbreviations

BC: Bronchogenic cyst
CT: Computed tomography
VATS: Video-assisted thoracic surgery
MRI: Magnetic resonance imaging
